# Macular Pigment Optical Density and Measures of Macular Function: Test-Retest Variability, Cross-Sectional Correlations, and Findings from the Zeaxanthin Pilot Study of Response to Supplementation (ZEASTRESS-Pilot)

**DOI:** 10.3390/foods5020032

**Published:** 2016-04-29

**Authors:** Alessandro Iannaccone, Giovannella Carboni, Gina Forma, Maria Giulia Mutolo, Barbara J. Jennings

**Affiliations:** 1 Hamilton Eye Institute, University of Tennessee Health Science Center, 930 Madison Avenue, Memphis, TN 38163, USA; carboni.gio@live.it (G.C.); gina.forma@gmail.com (G.F.); m_giulia9@hotmail.com (M.G.M.); math0618@bellsouth.net (B.J.J.); 2 Department of Ophthalmology, S. Giovanni di Dio Hospital, University of Cagliari, Via Ospedale, 86, Cagliari 09123, Italy; 3 Department of Ophthalmology, S. Andrea Hospital, II University Sapienza, Via di Grottarossa, 1035/1039, Rome 00189, Italy

**Keywords:** macular pigment optical density, zeaxanthin, pattern electroretinogram, contrast sensitivity

## Abstract

We report on the short-term test-retest baseline variability in macular function tests in ZEASTRESS-Pilot participants (*n* = 18), on their cross-sectional correlation with macular pigment optical density (MPOD), and on the effects of four months (FUV4) of 20 mg/day zeaxanthin (ZX), followed by a four-month washout (FUV8; *n* = 24, age 50–81 years old). Outcomes included: MPOD at 0.5 and 2.0 deg eccentricity (MPOD-0.5 and -2.0); contrast sensitivity (CS); pattern-reversal electroretinogram (PERG) amplitude; dark-adapted 650 nm foveal cone sensitivity (DA650-FCS); and 500 mn parafoveal rod sensitivity (DA500-PFRS). All measures of macular function showed close test-retest correlation (Pearson’s *r* range: 0.744–0.946) and low coefficients of variation (CV range: 1.13%–4.00%). MPOD correlated in a complex fashion with macular function. Following supplementation, MPOD-0.5 and MPOD-2.0 increased at *both* FUV4 and FUV8 (*p* ≤ 0.0001 for all measures). Continued, delayed MPOD increase and a small, but significant (*p* = 0.012), CS increase was seen at FUV8 only in females. PERGs increased significantly at FUV4 (*p* = 0.0006), followed by a partial decline at FUV8. In conclusion, following ZX supplementation, MPOD increased significantly. There was no effect on DA-650 FCS or DA-500 PFRS. Both CS and PERG amplitudes increased following supplementation, but the effect varied between males and females. Additional studies appear warranted to confirm and characterize further these inter-gender differences.

## 1. Introduction

The xantophylls lutein (LT), zeaxanthin (ZX), and meso-zeaxanthin (MZ) are the main *in vivo* biochemical determinants of macular pigment (MP). MP optical density (MPOD) is believed to have relevance in affecting macular visual performance to various visual stimuli and conditions [1,2,3,4,5,6,7,8,9,10,11,12,13,14,15,16], that these xanthophylls may act as singlet oxygen and free radical scavengers, and that MP may act as a blue light filter, protecting RPE and photoreceptors from light and oxidative damage [17,18,19,20]. Consistent, at least in part with these predictions, LT and ZX supplementation was effective in the AREDS2 trial in reducing the risk of progression to advanced disease in AMD [21], was shown to be preferable to the use of beta-carotene [22]. In addition to these macula-specific benefits of xanthophyll supplementation, interest in these carotenoids has significantly increased in the past few years because of other neural correlates and effects. For example, we have previously shown that MPOD is closely correlated with cognitive function in the elderly [23]. In this investigation, we showed that MPOD correlated better with cognitive function than serum levels of LT and ZX, suggesting that retinal *in vivo* levels of these carotenoids are a better proxy for actual neural uptake of LT and ZX than serum levels. This finding was consistent with the notion that LT and ZX account for a large proportion of the carotenoids found in the frontal and occipital cortex [24]. This MPOD-cognitive function correlation has been confirmed by several other investigators, and evidence has indeed been found for a direct relationship between retinal and brain LT/ZX levels at the histological level in both primates and human donor tissues [25,26,27,28,29,30,31]. In addition, there is now evidence also for a correlation between MPOD and auditory thresholds [32]. Therefore, there is much value in knowing which dietary supplements can be utilized to augment MPOD, what are its functional correlates, the extent of response to supplementation *in vivo*, and its dynamics.

While a considerable amount of data is available for LT supplementation, relatively little is known about MPOD changes following ZX supplementation. Van de Kraats *et al*. [33] investigated the effect of ZX-only supplementation on MPOD by fundus reflectometry in three male subjects, who took 20 mg ZX for six months and were followed for 10 months after supplementation. This study showed that, unlike participants on LT supplements, subjects on high-dose ZX experienced a sustained MPOD increase throughout the post-supplementation follow-up. This study suggested that response to high-dose ZX supplements may differ from that to high-dose LT. To our knowledge, a study like that of Van de Kraats *et al*. [33] has not yet been replicated, and no information is available on the response of women to high-dose dietary ZX.

In addition, with the exception of contrast sensitivity (CS), tolerance to photostress and multifocal electroretinogram (ERG) responses [1,2,3,4,5,6,7,8,9,10,11,12,13,14,15,16], there is limited information about the functional correlates of MPOD in normal subjects, either cross-sectionally or longitudinally, in response to supplementation. Understanding these correlations is important in order to have functional markers that could be used as indicators of macular benefit following supplementation that provide more refined insight than crude measurements of visual acuity, which is largely insensitive to supplementation especially in healthy subjects due mostly to a ceiling effect, and also to better comprehend how selective ZX supplementation could impact macular performance.

Our study aimed at beginning to provide this information, while also controlling for potential confounding from variables such as smoking, race [34,35,36,37,38,39,40], high body-mass index (BMI) at baseline, or BMI changes during the study (since high adiposity has been shown to correlate inversely with MPOD [41,42,43,44], likely acting as a sink for carotenoids ingested through diet). In order to achieve these objectives, we conducted the Zeaxanthin Pilot Study of Response to Supplementation (ZEASTRESS-Pilot) in healthy White participants, 50 to 85 years old, of either gender. In addition, we sought to also characterize at baseline the short-term test-retest variability of the outcomes measures of macular function that we investigated to facilitate the conduction of future larger supplementation studies based on these outcome measures, and investigated the correlation between them and MPOD both at baseline and following ZX supplementation.

With the caveat of its pilot nature, our study will show that the selected visual function outcome measures (as well as the MPOD measurements) exhibited excellent test-retest variability, that MPOD may correlate in a complex but functionally meaningful way with measures of macular function, that MPOD increases significantly after ZX supplementation with dynamics that differ from those of LT supplementation, and that there are potentially important inter-gender differences in the observed effects.

## 2. Materials and Methods

### 2.1. Subjects and ZEASTRESS-Pilot Study Design

The objective of this supplementation open-label study was to investigate the effect of a four-month, 20 mg/day ZX supplementation (two EyePromise-Ten gel capsules daily, generously provided by ZeaVision, St. Louis, MO, USA) followed by a four-month wash out period on MPOD in a group of White, healthy, non-smoking participants of BMI ≤ 30, between 50 and 85 years of age. Additional information about inclusion and exclusion criteria is provided below in Table 1. Participants underwent two baseline (BL) visits, termed Qualification Visit (QV) 1 and QV2, followed by daily 20 mg ZX supplementation, a four-month follow-up visit 4 (FUV4) and then after a four-month wash out period, leading up to a follow-up visit at eight months (FUV8). In between each four-month period, follow-up phone calls were performed by study staff at two (PC2) and six months (PC6) to ensure that participants were still remaining compliant with supplementation, record any changes in diet and lifestyle, adverse event, illness, problem, surgical procedure, or change in other circumstance that may have subsequently affected the interpretation of the study results.

### 2.2. Measurements

All study investigations were conducted at the Lions’ Visual Function Diagnostic Laboratory of the Hamilton Eye Institute, Univ. Tennessee Health Science Center (UTHSC) in Memphis, TN, USA. Informed written consent was obtained from all ZEASTRESS-Pilot participants, and the research was approved by the UTHSC Institutional Review Board (approval number: 09-00447-FB) and complied with the tenets of the Declaration of Helsinki. Data from the 24 enrolled healthy participants (F = 15; M = 9, age: 59.42 ± 7.37 years old; range: 50–81 years old) were analyzed in this study. Additional details about the inclusion and exclusion criteria used in this study are summarized in Table 1.

After a preliminary phone interview and obtaining fully-informed consent, prospective study participants underwent a series of screening measurements, which included anthropometric measurements of waist circumference (WC) and calculation of BMI according to standard techniques and equipment as reported in the literature and on the NIDDK website [46].

Visual acuity was measured at four meters with retroilluminated ETDRS standard charts 1 and 2 for the right and left eye, respectively [47], in the absence of sources of glare in a low photopic environment. Any eye not meeting the 20/25 entry criterion was refracted with chart R and any new/different best correction identified was used throughout the remainder of the study. ETDRS acuity was utilized in our study exclusively as an entry criterion, and no test-retest variability measurement was performed on this widely-established and routinely-utilized measure of visual function.

Following these screening measurements, eligible subjects underwent monocular CS testing according to standard methods with Pelli-Robson charts [48]. The number of letters read in each triplet was ascertained. Additional details are given in the Supplementary Methods section.

Following CS testing, MPOD was measured at 0.5 and 2.0 degs. of eccentricity from fixation (*vs*. a reference location at 7.0 deg of eccentricity) with a heterochromatic flicker photometry (HFP)-based instrument identical to the one developed by Wooten *et al*. [49] (Macular Metrics Corp., Rehoboth, MA, USA) and an elderly-friendly simplified methodology and testing protocol requiring only three repetitions, the details of which have been previously published [35], and the high reproducibility of which at the 0.5 deg testing location has already been documented [50]. Additional details on our testing methodology are illustrated in Supplementary Figures S1 and S2 and in the Supplementary Methods section.

Transient pattern ERG (PERG) macular responses were recorded with an Espion E3 electroretinography system (Diagnosys, Lowell, MA, USA), monocularly with a high-resolution CRT monitor with 45 min arc, 100% contrast pattern-reversal checks (see Supplementary Figure S3), in line with the 2007 ISCEV PERG standard and subsequent modifications [51,52]. The amplitude of the P50 positive PERG response was measured in µV as defined in the ISCEV standards [51,52], and used as the primary PERG outcome measure. An example of a PERG recording from one of the eyes of a study participants is shown in Supplementary Figure S3F.

Following pupil dilation and dark adaptation for 30 min, dark-adapted sensitivities were measured using Goldmann size-V stimuli with a Humphrey Field Analyzer (model 600 series; Zeiss-Humphrey Instruments, Dublin, CA, USA) custom-modified as previously reported [53,54] and previously utilized by our group [55,56,57,58]. Foveal dark-adapted cone-mediated sensitivity (DA650-FCS) was measured with a 650 -nm light target (Supplementary Figure S4A), and parafoveal (2 deg eccentricity) dark-adapted rod-mediated sensitivity (DA500-PFRS) was measured with an identical, 500 nm light target (Supplementary Figure S4B). These two locations were chosen for direct cross-correlation purposes with the eccentricity of the MPOD measurements. Additional details on our testing methodology are presented in the Supplementary Methods section.

Pelli-Robson CS, PERG, DA650-FCS, and DA500-PFRS measurements were repeated across two baseline (BL) visits, termed Qualification Visit (QV) 1 and QV2. From these measurements, the inter-visit correlation between measurements was assessed with linear regression and estimation of the Pearson’s coefficient of correlation, *r.* We then calculated the coefficient of variation (CV) for each outcome measure as an estimate of the short-term reproducibility of each test. Only data for subjects that could be retested within three weeks of each other were included in these test-retest variability analyses (*n* = 18; F/M = 11/7). This subgroup was slightly younger than the entire pool of participants, but not significantly so (range: 50–66; mean age: 58.39 ± 5.15 years, *p* > 0.05).

In this subgroup, we also investigated the BL correlation between MPOD and measures of macular function. For the purpose of these analyses, non-normally distributed data were natural log (Ln)-transformed. Correlations with DA650-FCS and DA500-PRFS were investigated only by MPOD location (0.5 and 2.0 deg. eccentricity, respectively). In addition, when investigating the relationship between MPOD and CS or PERG P50 amplitude, MPOD was also expressed as the sum of the values measured at 0.5 and 2.0 deg eccentricity (MPOD_tot_).

## 3. Results

### 3.1. Test-Retest Variability

All visual function tests displayed excellent reproducibility. CS was strongly correlated between QV1 and QV2 (Pearson’s *r* = 0.840) and the CV was 1.17% (± 2.03, SD). The intersession *r* for the PERG P50 amplitude was 0.946 and the CV for recordings conducted with our methodology was 4.00% ± 2.53%. DA650-FCS, and DA500-PFRS were also highly correlated between sessions (Pearson’s *r* = 0.744 and 0.908; CV: 2.29% ± 1.29% and 1.37% ± 1.13%, respectively).

MPOD test-retest variability was constrained to be <20% at the 0.5 deg eccentricity locus by study design as a requirement for enrollment after QV2 to ensure we could reliably ascertain the effects of supplementation on MPOD. With this caveat, MPOD-0.5 measurements displayed excellent reproducibility in all eligible ZEASTRESS-Pilot study participants, exhibiting a variability that was consistently <9% (CV: 3.83% ± 3.11%). This is even better a reproducibility than what we previously reported in an older population sample ≥ 70 years [50]. MPOD-2.0 measurements exhibited higher variability, but were nonetheless highly reproducible (CV: 10.57% ± 9.95%). Consistent with these findings, the inter-visit correlation between MPOD measurements was very high (Pearson’s *r* = 0.990 for MPOD-0.5 and 0.956 for MPOD-2.0). Based on these findings, the frequency of non-responders to ZX supplementation was also ascertained, and defined as participants who did not experience a percentage change (∆%) in the main MPOD outcome measure (MPOD-0.5) above the upper limit of 95% confidence interval for our observed CV (mean + 1.96 SD, *i.e.*, 9.92%).

### 3.2. Baseline Correlations

**Figure 1:**
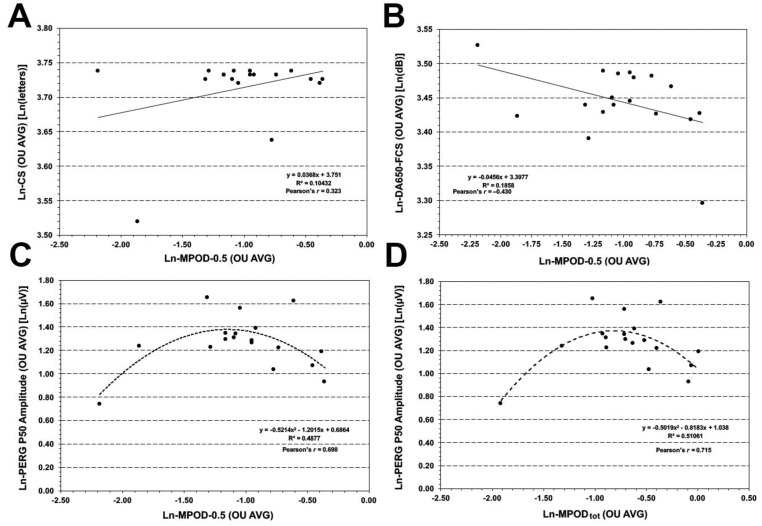
Baseline correlation analyses between macular pigment optical density (MPOD) and measures of macular function. The direct correlation between natural log (Ln) contrast sensitivity (CS) based on number of letters read at the Pelli-Robson test and Ln-MPOD measured at 0.5 deg of eccentricity (MPOD-0.5) is illustrated in (**A**). The inverse correlation between natural log (Ln) DA650-FCS and Ln-MPOD-0.5 is presented in (**B**). The curvilinear, ∩-shaped correlation between Ln-PERG P50 amplitude and Ln-MPOD-0.5 is shown in (**C**). The same correlation pattern was seen also for Ln-PERG P50 amplitude and Ln-MPOD_tot_ and is illustrated in (**D**).

Since measurements between eyes were highly inter-correlated, all BL correlations were investigated using the inter-ocular averages for each measured variable. As illustrated in Figure 1A, Ln-MPOD-0.5 was positively correlated to Ln-CS (*r* = 0.323). This correlation was considerably lower for Ln-MPOD-2.0 (*r*= 0.040, not shown) and, thus, also the correlation with Ln-MPOD_tot_ was diminished (*r* = 0.233, not shown) when Ln-MPOD-2.0 was averaged in. Ln-MPOD-0.5 showed an inverse relationship with DA650-FCS (*r* = −0.430, Figure 1B, whereas Ln-MPOD-2.0 was unrelated to DA500-PFRS (not shown). Interestingly, Ln-MPOD-0.5 displayed a curvilinear, ∩-shaped relationship with Ln-PERG P50 amplitude, with PERG P50 amplitudes tending to be largest for intermediate, and not the highest, MPOD levels. This ∩-shaped, curvilinear relationship was robust for both Ln-MPOD-0.5 (*r =* 0.698, Figure 1C) and Ln-MPOD_tot_ (*r =* 0.715, Figure 1D).

### 3.3. Post-Supplementation Findings

Only two participants who consented to take part in the ZEASTRESS-Pilot did not meet the qualification criteria at BL, and in both cases it was due to inability to perform MPOD testing within the required <20% test-retest variability between QV1 and QV2. A third participant who did complete QV1 and QV2 successfully and whose measurements were included in these short-term test-retest variability assessments was given the supplement but subsequently relocated unexpectedly and was unable to continue further participation in the ZEASTRESS-Pilot study and was, thus, excluded. With this exception, all other participants (*n* = 24) successfully completed the study and, with the exception of an occasional cold or bout of seasonal allergies, no participant suffered any adverse event in the course of the supplementation period either attributable or not attributable to the supplement, and no participant received any new prescription medication or started any new over-the-counter medication or supplement. In all 24 participants 20 mg ZX daily supplement compliance was ≥90% in all cases throughout the supplementation period. Compliance was ascertained both at the PC2 over the phone and personally at FUV4, in both instances by manual pill count. All results presented below are for these 24 participants, and all BL values reported herein are to be intended as the average of the measurements obtained at QV1 and QV2 on each participant.

No change in diet or lifestyle was ascertained in any of the participants in the course of the study, and BMI did not change during supplementation (BL mean ± 1 SD: 25.99 ± 2.52; FUV4: 26.26 ± 2.67; FUV8: 26.04 ± 2.61 *p* > 0.05 for both comparisons *vs*. BL). There was no significant correlation between the percent change (∆%) BMI and ∆% MPOD at either 0.5- or 2.0-deg. No participant developed any meaningful lens opacity in the course of the ~9 months during which the study was conducted.

**Figure 2:**
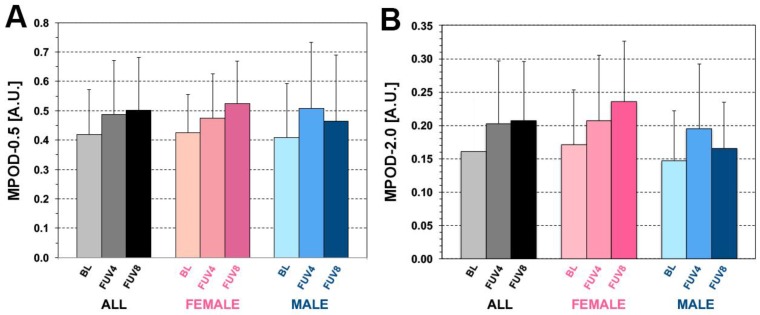
MPOD response to 20 mg/day ZX supplementation. The response to this supplementation regimen for MPOD-0.5 (foveal) and MPOD-2.0 (parafoveal) is illustrated in (**A**) and (**B**), respectively, for the entire study sample (grey-black bars) and for the female (pink, *n* = 15) and male (blue, *n* = 9) subgroups. Each bar shows the average ± 1 SD of the MPOD estimates obtained at each time point (baseline (BL), follow-up visit at four months (FUV4) and at eight months (FUV8)). Note the continued increase in MPOD at both locations at both time points, driven mainly by the female subgroup. The MPOD increase was marked and proportionally larger for the male subgroup at FUV4 but it was not followed by a sustained, delayed MPOD increase after wash-out, as seen in the female subgroup at FUV8.

As illustrated in Figure 2A, MPOD-0.5 increased significantly at both FUV4 and FUV8 (BL: 0.40 ± 0.17; FUV4: 0.49 ± 0.19; FUV8: 0.50 ± 0.17, *p*-value for paired *t*-test << 0.00001 for both). Significant increases occurred in both genders (mean ∆% at FUV4: F: +16%, M: +23%), continued further at FUV8 in females (mean ∆% at FUV8: +20%), but plateaued in males (+21%). As shown in Figure 2B, MPOD-2.0 showed the same trend (0.16 ± 0.08 at BL; 0.20 ± 0.10 at FUV4, *p* = 0.0001; 0.21 ± 0.09 at FUV8, *p* = 0.00002). Significant increases occurred in both genders (mean ∆% at FUV4: F: +12%, M: +20%) and continued further at FUV8 in females (mean ∆% at FUV8, F = +27%, *p* << 0.00001), whereas this phenomenon was not observed in males, in whom the observed MPOD-2.0 increases seen at FUV4 regressed partially (mean ∆% at FUV8, M = +11%, *p* > 0.05). At FUV4, we observed three non-responders. Two of these participants were females, one was male. These participants exhibited MPOD response at FUV8 (∆% values of +25.58%, +21.83%, and +13.45%, respectively). At FUV8, three participants who were responders at FUV4 (∆% values of +12.50%, +18.18%, and +22.50%, respectively) no longer met these criteria (∆% values *vs*. BL of +6.51%, +6.90%, and +3.13%, respectively) and had regressed to MPOD values within the BL variability levels. All other participants exhibited a response to supplementation at both time points.

On average, CS was unchanged at FUV4 *vs*. BL (whole study group: 40.97 ± 2.19 Pelli-Robson chart letters read at BL *vs*. 40.71 ± 2.82 at FUV4, *p* > 0.05). There was no detectable difference in CS between males and females at FUV4. This lack of change in CS for the study group as a whole persisted at FUV8 (41.22 ± 1.95 letters read, *p* > 0.05). However, consistent with the observed delayed effect on the MPOD, the female subgroup showed a biologically small, but statistically significant, CS increase *vs*. BL at FUV8 (40.37 ± 2.66 *vs*. 40.96 ± 2.44, *p*-value for paired, two-tailed *t*-test = 0.011). In ∆% terms, this change was equal to a positive CS change of 1.44% ± 2.89% for the female sub-group. The statistical significance of the finding is explained by the fact that only two measurements in the female subgroup exhibited a small reduction (−2.44% and −3.03%), while all others were either unchanged, or improved anywhere between 1.19% and 10.71%. No significant change was seen in CS for males at either point in time.

**Figure 3:**
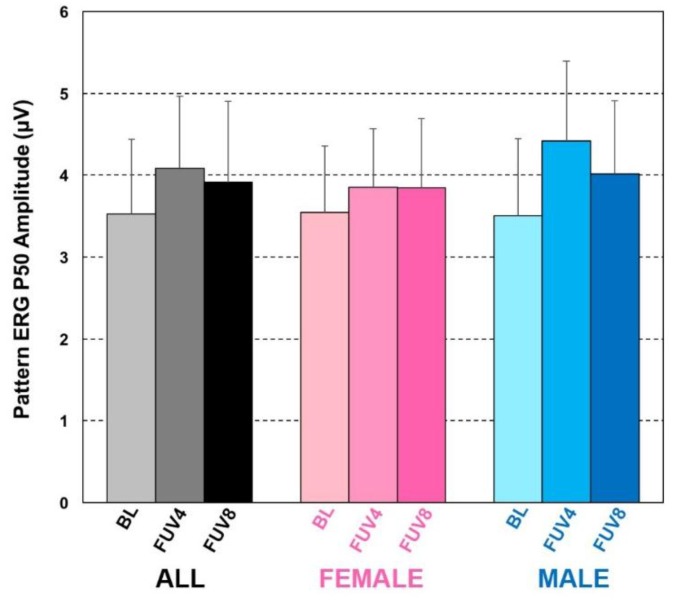
PERG P50 response to 20 mg/day ZX supplementation. Compared to BL, the PERG P50 response increased after supplementation for the whole study sample and in analyses stratified by gender. At FUV4, a 12% average PERG P50 increase was observed (*p* = 0.0002), followed only by a partial decline in this improvement after wash-out. Although small, this improvement remained significant *vs*. BL also at FUV8 (*p* = 0.01). The improvement in macular function at FUV4 by PERG P50 criteria was more robust in the male subgroup (*p* = 0.0014 at FUV4) than among females (*p* = 0.0473 at FUV4). Consistent with the persistent MPOD increases seen at FUV8, also PERG P50 amplitude remained elevated *vs*. BL at FUV8 in both subgroups, although this difference was no longer statistically significant.

Compared to BL (3.53 ± 0.91 µV), the PERG P50 response increased significantly after supplementation for the whole study sample and in analyses stratified by gender. At FUV4, a 12% average PERG P50 increase was observed (4.08 ± 0.81 µV, *p* = 0.0002), followed only by a partial decline in this improvement after wash-out (FUV8: 3.92 ± 0.94 µV, +6.8% on average *vs*. BL, Figure 3). Although small, because of the high reproducibility of our measurements and the paired data *t*-test analysis strategy, this improvement remained significant *vs*. BL also at FUV8 (*p* = 0.01). Different than what we observed for the MPOD and CS behaviors, the improvement in macular function at FUV4 by PERG P50 criteria was more robust in the male subgroup (3.51 ± 0.98 µV at BL; 4.42 ± 0.84 µV at FUV4, *p* = 0.0014) than among females (3.54 ± 0.88 µV at BL; 3.85 ± 0.72 µV at FUV4, *p* = 0.0473). Consistent with the persistent MPOD increases seen at FUV8, also PERG P50 amplitude remained elevated *vs*. BL at FUV8 in both subgroups (F: 3.85 ± 0.98 µV; M: 4.02 ± 0.89 µV), although this difference was no longer statistically significant (*p* > 0.05 in both cases).

Lastly, we observed no effect in either direction on DA-650 FCS after either four-month supplementation (BL: 31.3 ± 1.6 dB; FUV4: 31.0 ± 2.3 dB) or after washout (FUV8: 31.2 ± 2.2 dB). Likewise, there was no effect in either direction on DA-500 PFRS after either four-month supplementation (BL: 41.8 ± 2.6 dB; FUV4: 41.4 ± 2.6 dB) or after washout (FUV8: 41.9 ± 2.5 dB). Unlike what we observed for MPOD and CS values, there were no inter-gender differences in the behavior of dark-adapted sensitivities in our study sample.

## 4. Discussion

To the best of our knowledge, the ZEASTRESS-Pilot is the first study to report on the effects of selective high-dose (20 mg/day) ZX supplementation in a group of participants of both genders on both MPOD and measures of macular function, which provided us with insight on the biology of ZX retinal uptake and its possible beneficial effects, while controlling for potential confounding from smoking, race, high BMI at baseline, or BMI changes during the study. Van de Kraats *et al*. [33] investigated the effect of ZX-only supplementation on MPOD measured by fundus reflectometry in three male subjects, who took 20 mg/day ZX for six months and were followed for 10 months after supplementation. Their study showed that, unlike participants on LT supplements, subjects on ZX experienced a sustained MPOD increase throughout the post-supplementation follow-up. The ZEASTRESS-Pilot confirms the findings reported by this group in a larger sample size and extends them also to female participants, in which the effect was even more pronounced.

Characterization of the short-term test-retest BL variability of the chosen outcome measures provided us with useful information for future, larger-scale studies and trials of macular health and disease. CS assessed by Pelli-Robson charts, PERG P50 amplitudes, DA-650 FCS, and DA-500 PFRS as tested with our methods displayed excellent test-retest variability with average CVs ≤ 4% in all cases. Thus, these measurements appear all very well suited for studies of macular function and response to supplementation with ZX, similar carotenoids, and/or other agents that may improve macular visual performance. The same applies to MPOD measures which, although constrained by study design to have test-retest variability ≤ 20%, exhibited very low CV as well. The most reproducible of the measurements was the MPOD-0.5 (<9% CV in all cases, and mean CV of 3.83%). This reproducibility in the younger group that participated in the ZEASTRESS-Pilot using the elderly-friendly testing technique that we developed [35,50] is higher than what we previously reported for MPOD-0.5 in an older biracial population sample of participants ≥ 70 years old (*n* = 40, mean CV, 18.4%) [50]. However, in the latter sample, nearly 50% of the participants had a CV < 10%, indicating that age (and perhaps other factors) may be linked to higher test-retest variability in MPOD measures obtained with the HFP technique, but would not preclude meeting the ≤20% variability criterion that we had aimed for in this investigation. MPOD-2.0 measurements in the ZEASTRESS-Pilot participants exhibited higher variability, but were nonetheless highly reproducible as well (CV: 10.57% ± 9.95%). MPOD was not measured at this location in our earlier investigation; thus, we do not have an age-related term of comparison for MPOD-2.0, but it should be remembered that the ZEASTRESS-Pilot age range was 50–81 years. Although the short-term test-retest formal comparisons included a younger and smaller group (50–66 years, *n* = 18), inspection of the data for the older ZEASTRESS-Pilot participants that were among the ones who did not complete QV1 and QV2 within three weeks from one another (*n* = 2, ages 71 and 81 years old respectively), show little difference in test-retest variability compared to their younger peers (on average: 10.4% difference and as little as 2% for MPOD-0.5; and 9.3% difference and as little as 0%, *i.e.*, no difference, for MPOD-2.0). Thus, in summary, MPOD estimates can be obtained reliably at both 0.5 and 2.0 deg eccentricity locations.

Since MPOD is a strong correlate of cognitive function [23,24,25,26,30] and of the brain tissue levels of LT and ZX (and other related metabolites), this ability to provide reliable MPOD measurements with the HFP technique in subjects over a wide age range (*i.e.*, between 50 and 91 years old) provided by the present and our previous studies [35,50] has bearing also regarding possible investigations of the benefits of macular carotenoid supplementation towards improved cognitive aging in the elderly. This ZX-focused study may be especially important to this end, since a recent trial in Alzheimer’s disease (AD) with a daily combination of LT (10 mg), MZ (10 mg) and low-dose ZX (2 mg) did not show benefit on cognitive function in AD [31]. Thus, it is possible that a different combination of carotenoids and/or higher doses of ZX may be needed in AD to achieve therapeutic benefit, and/or that improved cognitive status may be attainable only in the context of an otherwise healthy cognitive aging but not in AD. Either way, our reproducibility estimates indicate that MPOD can be used reliably to gauge the amount of brain levels of the nutrients that contribute to MPOD augmentation. Accordingly, the CV estimates that we obtained in this study for the other measures of macular function can be used to calculate the sample sizes necessary to achieve statistical significance when testing hypotheses about the efficacy of any intervention in the future.

We also sought to begin better characterizing how MPOD correlates with macular function to gain insight on the functional implications of retinal MP content and on the benefits that could correlate with selective ZX supplementation. Our cross-sectional BL analyses suggest that higher MPOD levels may be associated with lower DA-650 FCS and comparisons at FUV4 and FUV8 suggested no effect on DA-650 FCS of a 20 mg daily ZX supplement. This finding was somewhat unexpected from a biological standpoint since we had previously reported in a case study of four-month 20 mg LT/0.9 mg ZX daily supplementation (Lutein caps, VitaminShoppe, North Bergen, NJ, USA) that DA-650 FCS improved significantly following supplementation [59]. The results of this experiment, which was conducted before the ZEASTRESS-Pilot, are presented in the Supplementary Results section and illustrated in Supplementary Figure S5 as a term of comparison. In that case study, MPOD increased following supplementation in both eyes (Supplementary Figure S5A,B) at all testing locations and, unlike ZX but consistent with previous studies of LT supplementation, MPOD declined back to or below BL levels after wash-out. These MPOD changes were accompanied by corresponding PERG P50 amplitude changes (Supplementary Figure S5C) in each eye. Further different than what we observed in the ZEASTRESS-Pilot, the 20 mg LT/0.9 mg ZX supplementation experiment led to a marked increase in DA650-FCS (Supplementary Figure S5D, inset). Consistent with the short-term effects of LT supplementation, after washout, DA650-FCS reverted to near-baseline levels.

With the caveat that our case study may have not been representative of all instances of LT supplementation, we used that information to guide our choices of outcome measures for the ZEASTRESS-Pilot and expected a 20 mg ZX supplement to mirror the functional outcomes we had seen with the 20 mg LT/0.9 mg ZX one. This was the case for PERG P50 amplitudes, but not for DA650-FCS, for which we also documented an inverse BL relationship with MPOD-0.5. The interpretation of this discrepancy between LT and ZX is uncertain. It is quite possible that it may reflect inherent differences in the biological effects of ZX and LT supplements at the retinal level, and this certainly warrants further investigation. It is also possible, however, that the different effect may reflect the different intra-retinal distribution of LT and ZX in the macular region. As Van de Kraats *et al*. [33] showed, LT has a flatter distribution, with a lower foveal peak and a broader distribution over the central macula, whereas ZX accumulates much more in the foveal region, with a higher foveal peak and a much steeper decline, extrafoveally. Thus, notwithstanding the aforementioned biological considerations, high-dose ZX supplements may impede the appreciation of any DA650-FCS benefit simply because of a more marked screening effect at the foveal level. These possibilities merit further investigation in larger studies.

The cross-sectional BL analyses that we performed between MPOD and measures of macular function also confirm that higher MPOD correlates with better CS, although it should be acknowledged that the correlation in our study sample appears to be driven primarily by a couple of subjects with low Ln-CS at baseline. It is possible that, in normal subjects, the actual extent of this correlation may be blunted by a ceiling effect. Additional studies on larger populations inclusive of subjects with macular pathology, like AMD, appear necessary to answer this question. Likewise, our findings also suggest that there may be an optimal MPOD range within which PERG amplitudes are highest. Curvilinear J-, U-, or ∩-shaped correlations are not uncommonly observed in human physiology. Thus, the latter finding is intriguing because it is uncertain as to whether the lower PERG P50 amplitudes in participants with MPOD levels at the highest end of normal reflect a blocking effect that denser MP may exert on optimal detection of high-contract pattern stimuli (which would potentially mesh well with the association between higher MPOD and better CS), whether it may reflect other yet poorly-understood intra-retinal neural implications of MPOD beyond the simple blue-light filtering effect, or a combination of the above. It will be of interest to study this phenomenon further in future, larger investigations.

Furthermore, consistent with our predictions, our previous experience with a 20 mg LT/0.9 mg ZX supplement [59], and the findings of Falsini *et al*. with steady-state focal macular ERG responses [60], PERG P50 amplitude exhibited an increase during active ZX supplementation. While the observed changes are, on average, not large, their direction was consistent and highly significant, providing proof of principle that ZX can also improve macular performance even in healthy subjects. Further studies on larger samples inclusive of patients with macular disease will be necessary to confirm and expand our findings from this pilot investigation. What was unexpected and not observed in response to the high LT/low ZX supplement [59,60] is that these PERG P50 amplitude improvements regressed only partially after discontinuation of ZX. However, this finding is perfectly in line with the observed behavior of MPOD and, thus, suggest that the residual PERG P50 increases may be linked at least in part to the persistently elevated MPOD.

Indeed, as expected, supplementation with 20 mg ZX increases significantly MPOD. As noted by van de Kraats *et al*., in three young male subjects [33], and unlike what seen by many with LT supplements, we confirmed that, following high-dose ZX supplements, MPOD increases are sustained well after wash-out. Our study expands the findings of van de Kraats *et al*. [33] confirming this effect in a larger participant sample. Our results also suggest that this unusual effect may be strongest in females, who experienced even larger MPOD increases after washout (FUV8) at both 0.5 and 2.0 deg. These delayed and sustained effects seen for ZX, and the observed M/F differences, were not expected. Thus, the study was not powered to address these subgroup analyses. With this caveat, these inter-gender differences may be due to distinct interactions with peripheral tissues (e.g., storage in, and release from fat, or in the corpus luteum in females), hormonal differences between males and females, inter-gender differences in the ability to store and/or retain ZX in the retina, both, or other yet unaccounted for factors. Inter-gender differences in the MPOD correlates have been noted previously [43]. Thus, larger investigations adequately powered to address these questions appear warranted.

Limitations of our investigation are inherent to its pilot study nature, and include: (a) the relatively small sample size and uneven M/F gender distribution—although this represents, to our knowledge, the largest high-dose ZX-only supplementation study to date, 24 participants may still not be representative of the entire gamut of the possible responses to ZX supplementation, whereby a larger study remains in order; (b) the open-label design of the ZEASTRESS-Pilot, whereby some form of bias may have been inadvertently introduced—a formal randomized, double-masked investigation would be warranted; (c) the fact that serum levels of ZX and/or other carotenoids were not measured—while supplement use compliance was excellent, it would have been informative to have information also on the serum response to supplementation in addition to the retinal MPOD levels (because of the sustained MPOD increase after discontinuation of the ZX supplement, this would have been especially valuable during the washout period); and (d) that neither microanatomical (e.g., macular spectral domain optical coherence tomography (SD-OCT)) nor genetic factors were investigated in our study.

The latter two factors may be very important in explaining the inter-gender differences suggested by the ZEASTRESS-Pilot, as well as inter-racial differences in MPOD distribution that we documented previously [35]. The relationship between MPOD and retinal thickness by SD-OCT criteria is ambiguous, with several studies that have documented an association with MPOD measured with the HFP technique [61,62,63,64,65], another in which the relationship varied between races [66], and at least one that used a fundus reflectance method that did not find a correlation [67]. Thus, it is possible that retinal thickness may be a better correlate of MPOD measured with a psychophysical method than with an imaging-based method. Additional studies inclusive of SD-OCT measures appear warranted.

It has been known for over a decade that MPOD is, at least in part, genetically determined [68] and correlates with foveal retinal thickness. Twin studies have confirmed the importance of genetic factors also in the MPOD response to supplementation [69]. In recent years, some of the genetic determinants of MPOD (variants in or near the *GSTP1, SCARB1, ABCA1, BCG5, LIPC, ELOVL2, FADS1, FADS2, ALDH3A2,* and *RPE65* genes) have been identified [70,71,72,73,74], leading Meyers *et al*. to conclude that “MPOD is a multi-factorial phenotype associated with variation in genes related to carotenoid transport, uptake, and metabolism, independent of known dietary and health influences on MPOD” [71]. While we fully share this view, in this study the authors also noted how the 13 SNPs from 10 genes that they found to be associated with MPOD accounted for only 5% of the variability in MPOD [71]. Thus, significant work remains to be done in this field and we suggest that future investigations of ZX supplementation, comparisons in MPOD response to selective ZX and LT supplements, and studies that addressed the inter-gender and inter-racial differences that we have observed in MPOD distribution and response to supplementation should take the opportunity to systematically investigate also the effect of genetic factors. The potential importance of the latter has already been established in the oncologic literature for the distribution of *GSTP1* alleles and their impact on both disease and response to treatment [75,76,77,78,79]. One can envision how genetic variants may also have a similar impact on MPOD augmentation and/or on the benefits of xanthophyll supplementation in AMD [21,22] or in cognitive aging.

Finally, whatever the explanation for the delayed and sustained effects of ZX supplementation that both van de Kraats *et al*. [33] and we documented, this phenomenon has very important implications for study design. Any study of high-dose ZX supplementation followed by a washout period and/or with a cross-over design must take into account the temporal dynamics of MPOD response to ZX supplementation and its difference from LT. Studies combining high or medium doses of both LT and ZX may be equally affected by the particular dynamics of the response to ZX supplementation. In addition, appropriate and well balanced study population stratification by gender appears in order, as are studies addressing the inter-gender differences that we observed, taking into account factors well beyond mere differences in genetic variants. In the ZEASTRESS-Pilot, Black participants were intentionally excluded to minimize the confounding that we expected to occur because of the interracial differences in MPOD that we have documented [35]. Thus, we suggest that these differences should be taken into account as well and future studies of MPOD supplementation should have appropriate and well balanced study population stratification also by race. In fact, much information about the biology of MPOD and response to xanthophyll supplementation may be derived from studies of bi- or multi-racial samples. Ultimately, as we make important strides towards personalized, precision medicine, improved understanding of the factors that affect susceptibility not only to disease, but also to the response to potentially-beneficial treatments is essential, and an understanding of who is likely to benefit from certain dietary supplements, or certain doses thereof, is an important and achievable goal.

## 5. Conclusions

In conclusion, following 4-month 20 mg ZX daily supplementation in normal subjects, MPOD increased significantly and, unlike LT, the observed effect was sustained also after the washout period. There was no effect of ZX on DA-650 FCS or DA-500 PFRS. A small increase in CS and a larger increase in PERG amplitudes was observed following ZX supplementation. Both the dynamics of MPOD increase and the effect on CS and PERG amplitudes appeared to differ between males and females. Additional studies appear warranted to confirm and characterize further these effects and to better understand the reasons for the observed inter-gender differences.

## Figures and Tables

**Table 1:** Inclusion and exclusion criteria for participation in ZEASTRESS-Pilot.

Inclusion criteria	Exclusion criteria
1. White/Caucasian ethnic background of both genders	1. Ethnic background other than White/Caucasian
2. Age 50 to 85 years old	2. Age < 50 years old or > 85 years old
3. Self-reported normal vision and/or absence of diagnosis of ocular disorders such as glaucoma, AMD, diabetic retinopathy, retinal vascular occlusions, inflammatory eye disorders such as uveitis, retinal detachment in both eyes at examination	3. Known diagnosis of, or presence at eye examination of inflammatory eye disease (e.g., uveitis, optic neuritis, papillitis), or known diagnosis of autoimmune disease and/or monoclonal gammopathy
4. Absence of autoimmune disease or monoclonal gammopathy	4. Body Mass Index (BMI) > 30
5. Best corrected visual acuity of 20/25 or better in at least one eye	5. Best corrected visual acuity < 20/25 in both eyes
6. Intraocular pressure (IOP) < 25 mmHg	6. IOP ≥ 25 mmHg in both eyes and/or established diagnosis of, or clear-cut signs of glaucoma
7. Dilatable pupils in at least one eye	7. Non-dilatable pupils, or unwillingness to undergo dilated exam
8. Clear ocular media in at least one eye based on AREDS criteria [45]	8. Dense media opacities in both eyes
9. Attached retina in at least one eye	9. History of current smoking
10. Ability and willingness to participate in the supplementation study for its entire duration, take the study supplements as directed, and take part in the eye exams and tests planned in the protocol	10. History of alcohol abuse or drug use
11. MPOD test-retest variability < 20% between baseline qualification visit 1 (QV1) and QV2	11. Use of dietary supplements containing ≥ 250 µg of LT or any amount of ZX (in order to minimize confounding introduced by the intake of other related or overlapping supplements)

## Supplementary Materials

The following are available online at www.mdpi.com/2072-6643/5/2/32/s1, Figure S1: Illustration of our HFP-based densitometry method, Figure S2: Illustration of the Excel-based interactive form used to calculate the MPOD for each participant, Figure S3: Illustration of the PERG methodology, Figure S4: Schematic illustration of the methodology used to measure dark-adapted sensitivities (DA650-FCS and DA500-PFRS), Figure S5: Summary of the results of a 20 mg LT/0.9 mg ZX supplementation case study.
