# Supplementary material for: Macular Pigment Optical Density and Measures of Macular Function: Test-Retest Variability, Cross-Sectional Correlations, and Findings from the Zeaxanthin Pilot Study of Response to Supplementation (ZEASTRESS-Pilot)
Source: Foods. 2016 Apr 29;5(2):32. doi: 10.3390/foods5020032 (PMC5302333; doi:10.3390/foods5020032)
Supplement: Supplementary file 1 [file foods-05-00032-s001.docx]

Supplementary Materials: Macular Pigment Optical Density and Measures of Macular Function: Test-Retest Variability, Cross-Sectional Correlations, and Findings from the Zeaxanthin Pilot Study of Response to Supplementation (ZEASTRESS-Pilot)

Alessandro Iannaccone, Giovannella Carboni, Gina Forma, Maria Giulia Mutolo and Barbara J. Jennings

1. Supplementary Methods

1.1. Pelli-Robson Chart Contrast Sensitivity (CS) Testing

Eligible subjects underwent monocular CS testing with Pelli-Robson charts [1] presented at three meters, with undilated pupils, in the absence of sources of glare under standard photopic conditions (room lighting conditions acceptable range 60–120 cd/m^2^). The luminance at the chart surface was measured with a Sekonic L-188 hand-held photometer before each testing session. The number of letters read in each triplet was ascertained. Within each triplet, all letters have the same contrast and contrast decreases from one triplet to the next. The test was interrupted when the participants could not read more than one letter in any given triplet, and the number of letters read correctly up to that point was recorded.

1.2. Macular Pigment Optical Density (MPOD) Testing

The method we used to measure MPOD was a psychophysical one based on heterochromatic flicker photometry (HFP) [2,3]. This method determines psychophysically the ratio between the visual sensitivity for a flickering test light that is maximally absorbed by MPs alternating in counterphase with a suppressing background at a wavelength that is not absorbed by the MP. The ratio between two measurements, one at the fovea and one parafoveally (where the MP density is minimal) provides an estimate of the MPOD.

Figure S1 illustrates the previously published testing method used in our study [4]. The appearance of the test targets from a participant’s perspective is illustrated in Figure S1A–C. To minimize test results’ variability and participants’ fatigue, we systematically instructed participants to identify the limits of the no-flicker zone for each target, which were termed the “minimum” and the “maximum” intensity values for the test target in question. This was done by adjusting first clockwise and then counter-clockwise the knob controlling the intensity of the test targets (Figure S1D), going always from flickering to not flickering and starting at the low intensity end (*i.e.*, minimums first, clockwise motion of the knob). Participants were always encouraged to blink several times when they first thought they reached a no-flicker point, and to continue adjusting the knob until the blinking no longer allowed the sensation of flickering in the test targets to resume. In the course of this study, the examiner performed the knob adjustment on behalf of all participants, instructing the subjects to notify immediately the examiner about cessation of flicker sensation in the test targets. A similar strategy has been used successfully also by others [5,6]. Once obtained, the minimum and maximum values were entered by the examiner in an Excel-based spreadsheet with embedded macros that calculated the exact mathematical average of these two numerical values (Figure S2), which identified the middle of the no-flicker zone, and entered it on the subjects’ behalf. This procedure was followed for all test targets for each of the study participants. In so doing, the middle of the no-flicker zone was the result of a precise average, rather than the subjective location thereof identified by the participant, and was attained faster than by letting the participants find it on their own by subsequent fine-tunings of the knob’s position.


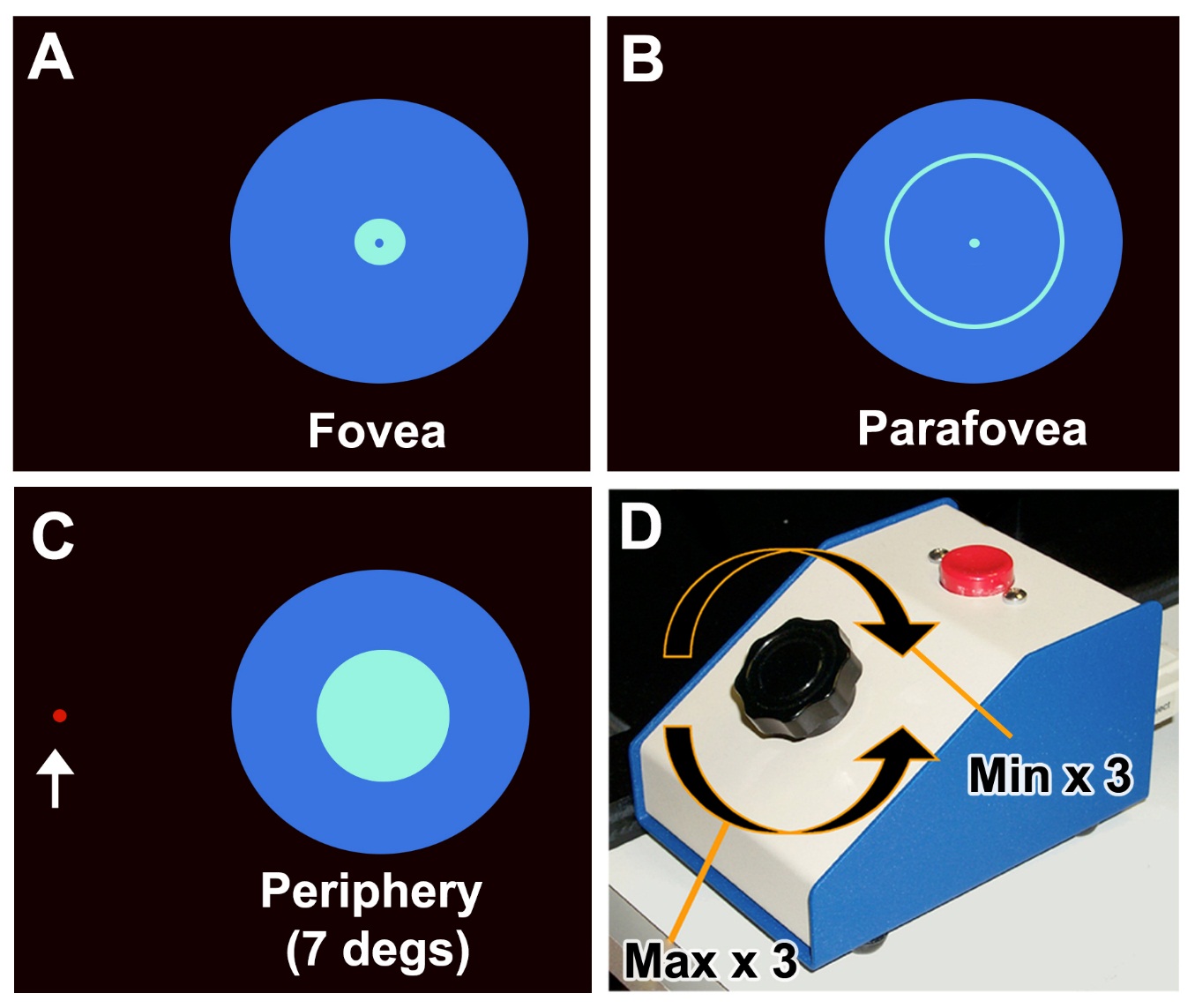


**Figure S1.** Illustration of our HFP-based densitometry method. Sketch of the appearance of the foveal test stimulus (0.5-deg eccentricity, or MPOD-0.5, (**A**)) and of the parafoveal flickering test stimulus (2.0-deg eccentricity, or MPOD-2.0, (**B**)) as seen from a participant’s perspective through the opening on the front of the instrument box. The 5-min target used for central fixation is shown in the middle of each stimulus; (**C**) Sketch of the appearance of the peripheral (7-deg eccentricity) stimulus towards which the 0.5 and 2.0-deg eccentricity measurements were compared to as reference. For this measurement, a lateral red LED-based fixation target is used, viewed through a side opening of the instrument. In each case, the test stimuli are shown surrounded by the suppressing background (see main text and previous publication of this methodology for further details); (**D**) Close-up view of the knob utilized to identify the lower and upper limits of the no-flicker zone: three pairs of clockwise (“minimums”) and counter-clockwise (“maximums”) progressive rotations of the knob were performed on behalf of the subject. The values were recorded and averaged.


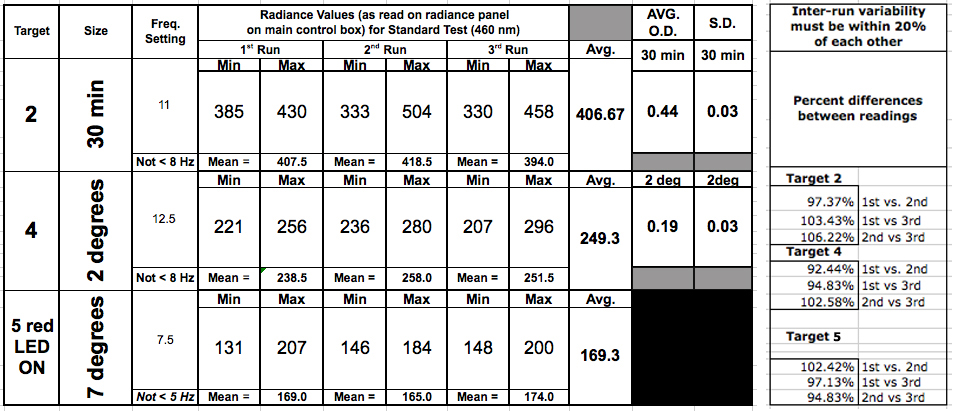


**Figure S2.** Illustration of the Excel-based interactive form used to calculate the MPOD for each participant. In this actual example (MPOD estimate from one of the ZEASTRESS-Pilot participants at baseline) the minimum and maximum values entered for each run and their individual averages are shown. The total average of the measurements is shown under the “Avg.” column in bold, and the MPOD estimates for each target (30 min, *i.e.*, 0.5-deg, and 2 deg) are shown to the right of the main panel, inclusive of their SD values. These values are calculated relative to the 7-deg average value (reference) taking into account a correction factor. To the far right, this interactive Excel-based form is completed by real-time calculations of the percent relationships between each run for each test target around the 100% value. In the example shown, for target 2 (0.5-deg, or 30 min) estimates varied between 3.43% and 6.22%, for target 4 (2.0-deg) between 2.58% and 7.36%, and for target 5 (7-deg reference measurement) between 2.42% and 5.17%.

1.3. Transient Pattern ERG (PERG) Methodology

Transient PERG macular responses were recorded with an Espion E3 ERG system (Diagnosys, Lowell, MA, USA), averaging at least 80–100 responses at each run, with HK-loop conjunctival electrodes [7] secured to the inferior eyelid skin as illustrated in Supplementary Figure S3A–D, following topical anesthesia with proparacaine HCl 0.5% and tetracaine HCl 0.5%. The covered fellow eye was used as the reference, and the right earlobe as ground. Responses were measured monocularly with a high-resolution CRT monitor with 45 min arc, 100% contrast pattern-reversal checks (Supplementary Figure S3E), alternating at 2-Hz, at the viewing distance of 1 m with best correction if required, and with undilated pupils meeting the 2007 ISCEV PERG standard and subsequent modifications [8,9]. Blink artifacts that were not automatically rejected were eliminated offline after the recording session. PERG amplitude was measured in µV from the trough of the N35 to the peak of the P50 component as per ISCEV standards [8,9] and expressed as the average of the two measurements (Supplementary Figure S3F) obtained at each session, and used as the primary PERG outcome measure.


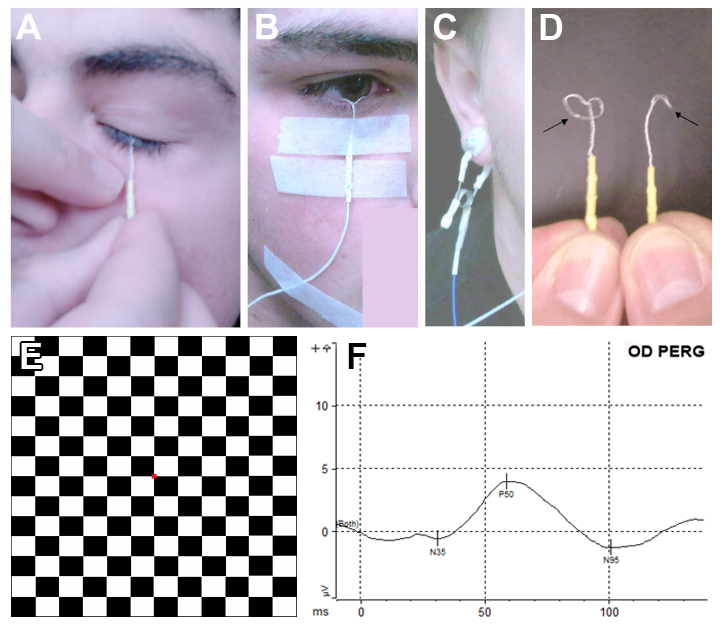


**Figure S3.** Illustration of the PERG methodology. After topical anesthesia, participants were first asked to look up, followed by gentle pulling down of the inferior eyelid and midline placement of the HK-loop electrode [7]. Once positioning was achieved, participants were asked to close gently their eyelids *(***A**); Following this step, three pieces of paper tape were secured to the inferior eyelid, cheek and malar area, holding in steady position the HK-loop electrode *(***B**); For recording purposes, each eye was tested separately, covering the fellow untested eye, using the HK-loop electrode to this eye as the reference. The ground electrode was always connected to the right earlobe *(***C**); The frontal and side appearance of the correctly prepared HK-loop electrode for a PERG recording session is shown in (**D**). The black arrow points to the recording portion of the HK-loop electrode, where three small electroconductive windows are present, facing towards the bulbar side of the conjunctiva. A schematic of a typical 45-min arc pattern-reversal stimulus used in the course of this investigation is shown in (**D**); Fixation was aided by a small red laser pointer light projected on the center of the CRT monitor, superimposed to the dim red square in the middle (generated by the monitor). An example of a transient (2-Hz) PERG response from the right eye (OD) of one of the participants is illustrated in (**E**). The amplitude of the P50 response was measured from the trough of the N35 to the peak of the P50 component as per ISCEV guidelines [8,9].

1.4. Dark-Adapted Foveal and Parafoveal Sensitivity Measurement Methodology

Following pupil dilation with 1% tropicamide and dark adaptation for 30 min, dark-adapted sensitivities were measured using Goldmann size-V stimuli of 200 ms duration with a Humphrey Field Analyzer (model 600 series; Zeiss-Humphrey Instruments, Dublin, CA, USA) custom-modified as previously reported [10,11] and previously utilized by our group [12–15]. Foveal dark-adapted cone-mediated sensitivity (DA650-FCS) was measured with a 650-nm stimulus (Supplementary Figure S4A), and parafoveal (2-deg eccentricity) dark-adapted rod-mediated sensitivity (DA500-PFRS) was measured with an identical, 500-nm stimulus presented two degs below a dim red-light fixation dot (Supplementary Figure S4B). Both DA650-FCS and DA500-PFRS were measured six times on each eye and the average of these measurements was taken as the point estimate of the sensitivities for each eye at each location. These two locations were chosen for direct cross-correlation purposes with the eccentricity of the MPOD measurements.


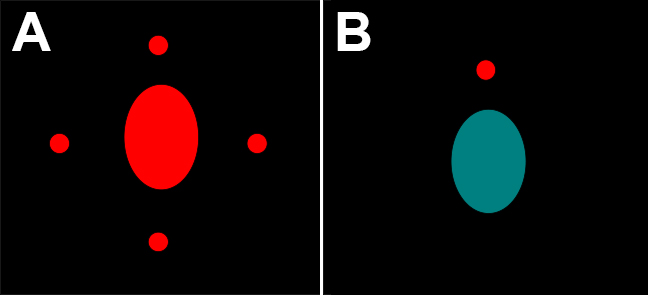


**Figure S4.** Schematic illustration of the methodology used to measure dark-adapted sensitivities (DA650-FCS and DA500-PFRS). Dark-adapted foveal cone sensitivities to 650 nm (red) stimuli (DA650-FCS) were measured with a size-V Goldmann stimulus presented centrally, in the middle of a fixation diamond comprised of 4 small red dots of dim light under fully DA conditions as shown in (**A**); Dark-adapted parafoveal rod sensitivities to 500 nm (blue-green) stimuli (DA500-PFRS) were measured under the same DA conditions using instead a single dim central red fixation dot, presenting the 500 nm stimulus of the same size and shape 2-degs inferiorly to the fixation dot, as shown in (**B**).

2. Supplementary Results

Our finding of a lack of effect of 20 mg ZX supplementation on DA650-FCS was somewhat unexpected from a biological standpoint since we had previously reported in a case study of 4-month 20 mg LT/0.9 mg ZX daily supplementation (Lutein caps, VitaminShoppe, North Bergen, NJ, USA) that DA-650 FCS improved significantly following supplementation [16]. The results of this experiment, which was conducted before ZEASTRESS-Pilot, are summarized below, and include previously unreported follow-up data following a 4-month wash-out period after supplementation.

In this case study, conducted on a White, male, 21-year old healthy subject, following supplementation MPOD increased in both eyes (Supplementary Figure S5A,B) at all testing locations, including the 0.5- and 2.0-deg. locations used in the present investigation (red dashed boxes) and, unlike ZX but consistent with previous studies of LT supplementation, MPOD declined back to or below BL levels after the wash-out period. These MPOD changes were accompanied by corresponding PERG P50 amplitude changes (Supplementary Figure S5C) in each eye. This included a “negative rebound” below BL levels that we did not observe in ZEASTRESS-Pilot following ZX supplementation. Further different than what we observed in ZEASTRESS-Pilot, the 20 mg LT/0.9 mg ZX supplementation experiment led to a marked increase in DA650-FCS (Supplementary Figure S5D, inset). Consistent with the short-term effects of LT supplementation, after wash-out DA650-FCS reverted to near-baseline levels. Of interest, in this case study we also investigated the kinetics of cone recovery in response to the same DA650-FCS stimulus after a full (97%) rhodopsin bleach as used for conventional dark adaptometry and we observed no effect of the 20 mg LT/0.9 mg ZX supplement on the kinetics of cone recovery (Supplementary Figure S5D). Because of this lack of impact of the high dose LT supplement on this parameter, the latter was not included in the ZEASTRESS-Pilot study.


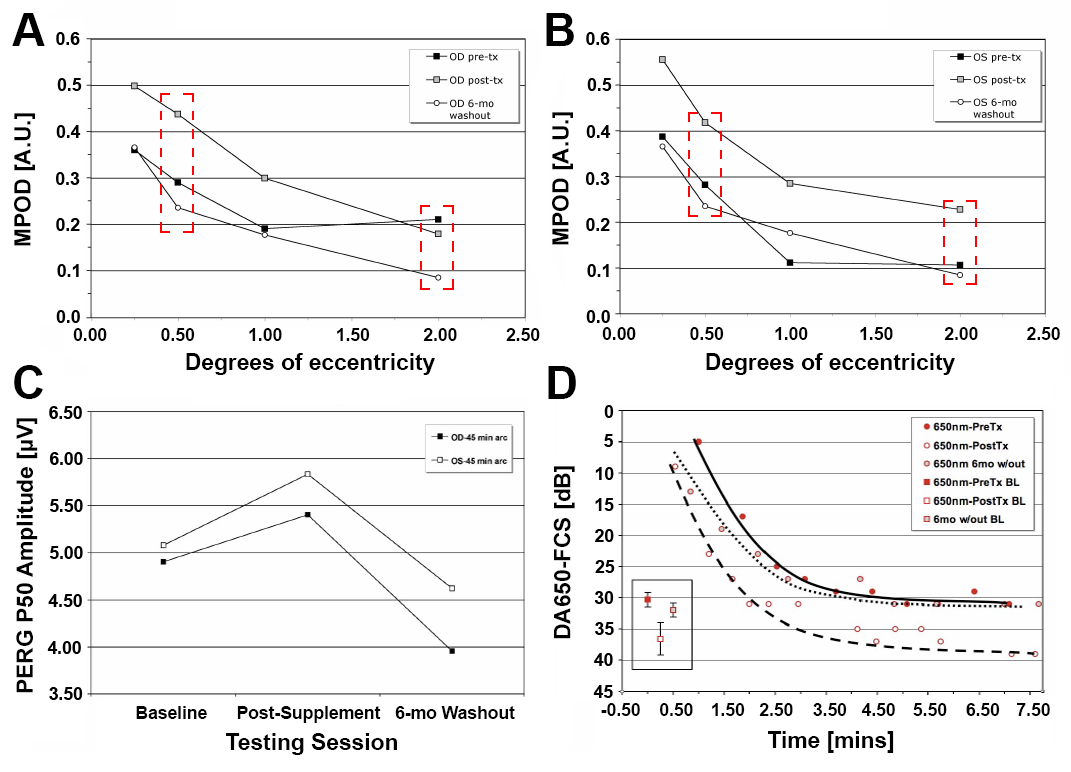


**Figure S5.** Summary of the results of a 20 mg LT/0.9 mg ZX supplementation case study. The results of this case study are presented as a term of comparison to the 20 mg ZX supplementation data. The MPOD response in this experiment for the right (OD) and left eye (OS) are presented in (**A**) and (**B**), respectively. MPOD was measured in the case study at 4 locations instead of the two used in the ZEASTRESS-Pilot study. Thus, the two corresponding locations (MPOD-0.5 and MPOD-2.0) are highlighted in (**A**) and (**B**) by red dashed boxes for easier comparison; The changes in PERG P50 amplitude are illustrated for both eyes in (**C**); Notice the parallel between the negative rebound in MPOD levels after wash-out and the same phenomenon occurring for PERG P50 amplitudes. DA650-FCS results from this case study are shown in (**D**). The fully dark-adapted sensitivities measured precisely as in the ZEASTRESS-Pilot study are presented in the inset for pre-treatment (BL), post-treatment, and washout testing session. In addition, **(D**) illustrates the recovery of the DA650-FCS following a full retinal bleach (cone branch of the dark adaptation curve). While final sensitivities at cone plateau were shifted down (*i.e.*, were improved) following supplementation, the overall kinetics of DA650-FCS recovery (shown by the dashed best-fit curves) were not visibly affected by the 20 mg LT/0.9 mg ZX supplement.

References

1. Pelli, D.G.; Robson, J.G.; Wilkins, A.J. The design of a new letter chart for measuring contrast sensitivity*.* *Clin. Vis. Sci.* **1988**, *2*, 187–199.
2. Snodderly, D.M.; Hammond, B.R., Jr. *In vivo* psychophysical assessment of nutritional and environmental influences on human ocular tissues: Lens and macular pigment*.* In *Nutritional and Environmental Influences on the Eye*; Taylor, A.J., Ed.; CRC Press: Boca Raton, FL, USA, 1999; pp. 251–273.
3. Bone, R.A.; Landrum, J.T. Heterochromatic flicker photometry*.* *Arch. Biochem. Biophys.* **2004**, *430*, 137–142.
4. Iannaccone, A.; Mura, M.; Gallaher, K.T.; Johnson, E.J.; Todd, W.A.; Kenyon, E.; Harris, T.L.; Harris, T.; Satterfield, S.; Johnson, K.C.; *et al*. Macular pigment optical density in the elderly: Findings in a large biracial mid-south sample*.* *Investig. Ophthalmol. Vis. Sci.* **2007**, *48*, 1458–1465.
5. Aleman, T.S.; Duncan, J.L.; Bieber, M.L.; de Castro, E.; Marks, D.A.; Gardner, L.M.; Steinberg, J.D.; Cideciyan, A.V.; Maguire, M.G.; Jacobson, S.G. Macular pigment and lutein supplementation in retinitis pigmentosa and usher syndrome*.* *Investig. Ophthalmol. Vis. Sci.* **2001**, *42*, 1873–1881.
6. Duncan, J.L.; Aleman, T.S.; Gardner, L.M.; De Castro, E.; Marks, D.A.; Emmons, J.M.; Bieber, M.L.; Steinberg, J.D.; Bennett, J.; Stone, E.M.; *et al*. Macular pigment and lutein supplementation in choroideremia*.* *Exp. Eye Res.* **2002**, *74*, 371–381.
7. Hawlina, M.; Konec, B. New noncorneal HK-loop electrode for clinical electroretinography*.* *Doc. Ophthalmol.* **1992**, *81*, 253–259.
8. Holder, G.E.; Brigell, M.G.; Hawlina, M.; Meigen, T.; Vaegan; Bach, M. ISCEV standard for clinical pattern electroretinography—2007 update*.* *Doc. Ophthalmol.* **2007**, *114*, 111–116.
9. Bach, M.; Brigell, M.G.; Hawlina, M.; Holder, G.E.; Johnson, M.A.; McCulloch, D.L.; Meigen, T.; Viswanathan, S. ISCEV standard for clinical pattern electroretinography (PERG): 2012 update*.* *Doc. Ophthalmol.* **2013**, *126*, 1–7.
10. Apáthy, P.P.; Jacobson, S.G.; Nghiem-Phu, L.; Knighton, R.W.; Parel, J.-M. Computer-aided analysis in automated dark-adapted static perimetry. In [Seventh International Visual Field Symposium, Amsterdam, September 1986](http://link.springer.com/book/10.1007/978-94-009-3325-5); Springer: Dordrecht, Netherlands, 1987; Volume 49, pp. 277–284.
11. Jacobson, S.G.; Voigt, W.J.; Parel, J.-M.; Apathy, P.P.; Nghiem-Phu, L.; Myers, S.W.; Patella, V.M. Automated light- and dark-adapted perimetry for evaluating retinitis pigmentosa. *Ophthalmology* **1986**, *93*, 1604–1611.
12. Jacobson, S.G.; Cideciyan, A.V.; Iannaccone, A.; Weleber, R.G.; Fishman, G.A.; Maguire, A.M.; Affatigato, L.M.; Bennett, J.; Pierce, E.A.; Danciger, M.; *et al*. Disease Expression of *RP1* mutations causing autosomal dominant retinitis pigmentosa. *Investig. Ophthalmol. Vis. Sci.* **2000**, *41*, 1898–1908.
13. Iannaccone, A.; Man, D.; Waseem, N.; [Jennings, B.J](http://www.ncbi.nlm.nih.gov/pubmed/?term=Jennings%20BJ%5BAuthor%5D&cauthor=true&cauthor_uid=17014888).; [Ganapathiraju, M](http://www.ncbi.nlm.nih.gov/pubmed/?term=Ganapathiraju%20M%5BAuthor%5D&cauthor=true&cauthor_uid=17014888).; [Gallaher, K](http://www.ncbi.nlm.nih.gov/pubmed/?term=Gallaher%20K%5BAuthor%5D&cauthor=true&cauthor_uid=17014888).; [Reese, E](http://www.ncbi.nlm.nih.gov/pubmed/?term=Reese%20E%5BAuthor%5D&cauthor=true&cauthor_uid=17014888).; [Bhattacharya, S.S](http://www.ncbi.nlm.nih.gov/pubmed/?term=Bhattacharya%20SS%5BAuthor%5D&cauthor=true&cauthor_uid=17014888).; [Klein-Seetharaman, J](http://www.ncbi.nlm.nih.gov/pubmed/?term=Klein-Seetharaman%20J%5BAuthor%5D&cauthor=true&cauthor_uid=17014888). Retinitis Pigmentosa Associated with Rhodopsin Mutations: Correlation between Phenotypic Variability and Molecular Effects*.* *Vision Res.* **2006**, *46*, 4556–4567.
14. Mura, M.; Sereda, C.; Jablonski, M.M.; MacDonald, I.M.; Iannaccone, A. Clinical and functional findings in choroideremia due to complete deletion of the *CHM* gene*.* *Arch. Ophthalmol.* **2007**, *125*, 1107–1113.
15. Iannaccone, A.; Othman, M.I.; Cantrell, A.D.; Jennings, B.J.; Branham, K.; Swaroop, A. Retinal phenotype of an X-linked pseudo-Usher syndrome in association with the G173R mutation in the *RPGR* gene*.* *Adv. Exp. Med. Biol.* **2008**, *613*, 221–227.
16. [Bosco, A](http://www.ncbi.nlm.nih.gov/pubmed/?term=Bosco%20A%5BAuthor%5D&cauthor=true&cauthor_uid=18385061).; [Inman, D.M](http://www.ncbi.nlm.nih.gov/pubmed/?term=Inman%20DM%5BAuthor%5D&cauthor=true&cauthor_uid=18385061).; [Steele, M.R](http://www.ncbi.nlm.nih.gov/pubmed/?term=Steele%20MR%5BAuthor%5D&cauthor=true&cauthor_uid=18385061).; [Wu, G](http://www.ncbi.nlm.nih.gov/pubmed/?term=Wu%20G%5BAuthor%5D&cauthor=true&cauthor_uid=18385061).; [Soto, I](http://www.ncbi.nlm.nih.gov/pubmed/?term=Soto%20I%5BAuthor%5D&cauthor=true&cauthor_uid=18385061).; [Marsh-Armstrong, N](http://www.ncbi.nlm.nih.gov/pubmed/?term=Marsh-Armstrong%20N%5BAuthor%5D&cauthor=true&cauthor_uid=18385061).; [Hubbard, W.C](http://www.ncbi.nlm.nih.gov/pubmed/?term=Hubbard%20WC%5BAuthor%5D&cauthor=true&cauthor_uid=18385061).; [Calkins, D.J](http://www.ncbi.nlm.nih.gov/pubmed/?term=Calkins%20DJ%5BAuthor%5D&cauthor=true&cauthor_uid=18385061).; [Horner, P.J](http://www.ncbi.nlm.nih.gov/pubmed/?term=Horner%20PJ%5BAuthor%5D&cauthor=true&cauthor_uid=18385061).; [Vetter, M.L](http://www.ncbi.nlm.nih.gov/pubmed/?term=Vetter%20ML%5BAuthor%5D&cauthor=true&cauthor_uid=18385061). Reduced retina microglial activation and improved optic nerve integrity with minocycline treatment in the DBA/2J mouse model of glaucoma. *Investig. Ophthalmol. Vis. Sci.* **2008**, *48*, 1437–1446.
